# Evolution of policies on human resources for health: opportunities and constraints in four post-conflict and post-crisis settings

**DOI:** 10.1186/s13031-016-0099-0

**Published:** 2017-01-18

**Authors:** Sophie Witter, Maria Paola Bertone, Yotamu Chirwa, Justine Namakula, Sovannarith So, Haja R. Wurie

**Affiliations:** 1 ReBUILD and Institute for Global Health and Development, Queen Margaret University, Edinburgh, EH21 6UU UK; 2 Department of Global Health and Development & ReBUILD Consortium, London School of Hygiene and Tropical Medicine, London, UK; 3 ReBUILD and Biomedical Research and Training Institute, Harare, Zimbabwe; 4 ReBUILD and Department of Health Policy, Planning and Management, Makerere School of Public Health, Kampala, Uganda; 5 ReBUILD and Cambodian Development Resource Institute, Phnom Penh, Cambodia; 6 ReBUILD Consortium and College of Medicine and Allied Health Sciences, University of Sierra Leone, Freetown, Sierra Leone

**Keywords:** Health workers, Post-conflict, Human resources for health, Policy analysis, Uganda, Zimbabwe, Cambodia, Sierra Leone

## Abstract

**Background:**

Few studies look at policy making in the health sector in the aftermath of a conflict or crisis and even fewer specifically focus on Human Resources for Health, which is a critical domain for health sector performance. The main objective of the article is to shed light on the patterns and drivers of post-conflict policy-making. In particular, we explore whether the post -conflict period offers increased chances for the opening of ‘windows for opportunity’ for change and reform and the potential to reset health systems.

**Methods:**

This article uses a comparative policy analysis framework. It is based on qualitative data, collected using three main tools - stakeholder mapping, key informant interviews and document reviews - in Uganda, Sierra Leone, Cambodia and Zimbabwe.

**Results:**

We found that HRH challenges were widely shared across the four cases in the post-conflict period but that the policy trajectories were different – driven by the nature of the conflicts but also the wider context. Our findings suggest that there is no formula for whether or when a ‘window of opportunity’ will arise which allows health systems to be reset. Problems are well understood in all four cases but core issues – such as adequate pay, effective distribution and HRH management – are to a greater or lesser degree unresolved. These problems are not confined to post-conflict settings, but underlying challenges to addressing them – including fiscal space, political consensus, willingness to pursue public objectives over private, and personal and institutional capacity to manage technical solutions – are liable to be even more acute in these settings. The role of the MoH emerged as weaker than expected, while the shift from donor dependence was clearly not linear and can take a considerable time.

**Conclusions:**

Windows of opportunity for change and reform can occur but are by no means guaranteed by a crisis – rather they depend on a constellation of leadership, financing, and capacity. Recognition of urgency is certainly a facilitator but not sufficient alone. Post-conflict environments face particularly severe challenges to evidence-based policy making and policy implementation, which also constrain their ability to effectively use the windows which are presented.

## Background

Health worker attraction, retention, distribution and performance are arguably the most critical factors affecting the performance of a health system [1]. In post-conflict and post-crisis settings where health systems and health worker livelihoods have been disrupted, the challenges facing the establishment of the right working environment are particularly important, and the contextual dynamics around them extremely relevant to understand and incorporate sensitively into policy measures [2]. Few studies look at policy making in the health sector in the aftermath of a conflict or crisis [3, 4] and even fewer specifically focus on Human Resources for Health (HRH) [5, 6], making HRH policies in post-conflict settings an overall under researched topic. Moreover, most of the existing studies focus on the policies per se [7], rather than at the policy- making trajectory, which is the core subject of this study.

In this article, we extend the findings of the literature on policy making in post-conflict and post-crisis by analysing across a set of case studies. The main objective of the article is to shed light on the patterns and drivers of policy-making post-conflict and post-crisis. In particular, we explore whether this period offers increased chances for the opening of ‘windows of opportunity’ for change and reform and the potential to reset health systems [8]. Given this objective, our main focus is specifically on the policy processes and the drivers of the policy design, as well as their content and the outcomes of their implementation. Although our attention is predominantly on the policies regarding HRH, our findings also touch elements concerning the broader health system, its financing and organization, which are often intertwined. Moreover, we believe that the analysis of the policy-making trajectory and drivers for HRH issues offers insights which can illuminate the general features and specificities of policy-making processes in post-conflict and post-crisis health systems.

This article builds on the systematic comparison of case studies carried out in four countries, under the ReBUILD research project. ReBUILD is a Department for International Development (DfID)-funded research partnership, whose work spanned from 2011 to 2016 and aims to produce a body of relevant research on health systems in post-conflict settings. Specifically, the four study settings are Cambodia, (northern) Uganda, Sierra Leone and Zimbabwe. The choice of the four countries allows us to cover a range of different conflict or crisis and post-conflict/crisis experiences, which are further described in the context section below and which include countries at different stages of distance from recent conflicts.

It is important to note that, although we often use the term ‘post-conflict’ for sake of simplicity throughout this article, our research is carried out in one post-crisis environment (Zimbabwe). In relation to Uganda, although we use Uganda and northern Uganda interchangeably, the main study setting was northern Uganda, the region which experienced protracted conflict. Additionally, we use the term ‘policy’ in a very broad sense, including not only official policies and strategies on HRH, but also programmatic changes and initiatives that affected health workers, which were approved in an alternative form to an official policy document. These are important as in post-conflict situations some of the most significant changes may come from these less formal sources.

We start by presenting a brief background on the conflicts and crises in these four settings, followed by our methods. We then use a health policy analysis framework to examine the HRH challenges which arose post-conflict and how the policy trajectory in each case responded to them, along with the actors and factors which influenced policy change. Finally, we examine how effective the policies have been in addressing HRH challenges. All of these feed into a final discussion of what we can learn about policy opportunities and constraints in post-conflict settings.

### Four country contexts

In northern Uganda, the prolonged and widespread insurgency lasted 20 years (1986–2006) and displaced populations across the region [9] while the rest of the country remained largely peaceful. The conflict has profoundly affected the economic and social fabric of the area and had a deeply negative impact for the broader health system. In terms of HRH, the changes were stark as the majority of health workers fled to safer places whereas those who stayed behind were traumatized, struggled to cope with worsened working conditions among other hindrances, and often narrowly survived death [10]. In 2006, the Lord’s Resistance Army (LRA) was expelled from the region and peace talks began. The ceasefire was followed by efforts to resettle the populations to their home villages. The early post-conflict period also led to the implementation of various recovery activities under the Peace Recovery and Development Plan (PRDP 2007) and aid donations aimed to improve the general health service delivery in the affected parts of the northern region [11].

In Sierra Leone, the conflict dates from March 1991 when rebels of the Revolutionary United Front launched an attack from the east of the country near the border with Liberia to overthrow the government. The resulting civil war spanned 11 years, ending in 2002, when new elections were held. During the war time, it is estimated that over 50,000 people were killed and 2 million displaced, which amounted to almost half of the population [12]. The war also devastated the healthcare system. The vast majority of the health infrastructure was destroyed, and health worker attrition rates increased, which compromised efforts to provide equitable access to health care in the aftermath of the conflict [13]. A decade after the war, Sierra Leone still suffers from the effects of the conflict, and the gains made over time to strengthen the health sector were subject to a major setback as a result of the 2014 Ebola outbreak [14].

In Cambodia, the conflict dates back to the US carpet bombing during the Vietnam War (starting in 1969) and the imposition of Lon Nol to prevent Cambodian support for Vietnam. Internal political conflicts led to a military coup in March 1970 which brought in Vietnamese and United States involvement. The consequence was a radical insurgency called the Khmer Rouge, which took over power in 1975–1978. The Khmer Rouge destroyed all social and economic infrastructures with the aim of turning Cambodia into an agrarian society. In the process, about two million people died from starvation, diseases, execution and institutional destruction [15]. The Khmer Rouge was partially overthrown in 1979 by the Vietnamese who formed the People’s Republic of Kampuchea (PRK) and helped reconstruct the state institutions based on a socialist ideology. However, civil war continued between the government and the remnants of the Khmer Rouge, until a peace agreement was reached in 1991. The national election in 1993 sponsored by UN was held successfully, but factional fighting broke out again in 1997 [16] and peace as a realistic description of the situation can only be dated to early 1999 after the death of the Khmer Rouge leader Pol Pot. At that time, a process of political and economic liberalisation of the country took place, and international aid was critical to support the country’s reconstruction. Although the end of the conflict is now almost two decades back, the challenges for the health system remain stark to this day [17].

Although Zimbabwe experienced conflict during its war of independence in the 1980s, its more recent history has been characterized by a period of severe economic, social and political crisis between 1997 and 2009. The decade-long socio-economic crisis caused the decline of Zimbabwe’s Gross Domestic Product (GDP), leading to constrained capability to finance government services. Between 2000 and 2009, Zimbabwe’s real GDP declined by 5.9 % annually.. Cumulatively, output declined by more than 40 % between 2000 and 2007 [18]. The economy experienced high inflation between 2000 and 2008. By mid-2008, hyper-inflation led to the demonetisation of the Zimbabwe dollar and the adoption of multiple currencies as official tender in 2009. A marked decline in living standards and increase in poverty occurred during this crisis period.

From 2005, the health system experienced sharp decreases in funding and health spending dropped to a mere 0.3 % of the entire national budget. This resulted in the deterioration of health infrastructure, loss of experienced health professionals, drug shortages, increased burden of disease and the attendant high demand for services [19]. The crisis abated when a coalition government was formed between the two major parties, the Zimbabwe African Union Patriotic Front and the Movement for Democratic Change. This enabled the provision of financial support for various government programs by the development partners [18].

## Methods

This study is based on a comparative policy analysis framework, focusing on the exploration of the HRH policy-making processes, the policy patterns, the key elements driving them and the consequences for the policy implementation. This is used to investigate the proposition that the immediate post-conflict period offers a ‘window of opportunity’ to reset trajectories within the health system.

### Data tools and collection

This article makes use of mostly qualitative data, collected between 2012 and 2013, in the four countries using three main tools: a stakeholder mapping (in two of the countries), and key informant interviews and a document review in all four contexts (Table 1).

**Table 1:** Overview of data collection methods by country

	Cambodia	Uganda	Sierra Leone	Zimbabwe
Document review	√	√	√	√
*(reference period for the document search)*	(1979-present)	(1999–2014)	(2000–2012)	(1997–2012)
*(num. of documents retrieved)*	(59)	(59)	(76)	(76)
Key informant interviews (KII) *(n)*	√ (33)	√ (25)	√ (23)	√ (28)
Stakeholder mapping		√	√	

In Uganda and Sierra Leone, a *stakeholder mapping* exercise took place, bringing together key stakeholders at national level (and at regional/sub-national level in Uganda) to discuss the role of the main actors who had influenced HRH policy and practice over the different periods. The meeting was facilitated by the research team and the participants were asked about their narratives of the HRH policy-making processes and, in particular, to map all the actors concerned (whether present or not), for their influence and interest in HRH issues on a 1–5 scale. The stakeholder meeting was carried out prior to the rest of the data collection and played an important role in providing a better understanding of the overall context in the countries in terms of HRH and identifying key informants who were then contacted for the interviews. The exercise was not conducted in Cambodia and Zimbabwe as the group exercise was considered unsuitable for this potentially sensitive activity in these contexts.

In all study settings, a thorough *review of the documentation* available was carried out, including both grey and published literature. The focus was on the HRH documents, including policies, strategies, reports and evaluations, as well as general health sector policies useful to provide a detailed background. Documents from governmental sources (Ministry of Health, as well as other Ministries and the Office of the President), but also from development partners, donors and NGOs, local print media, civil society and academic researchers were included in the search and review. In some cases, the document search also comprised secondary data and databases. The time reference for the document review varied in each country, but focussed on the periods during and post-conflict, where the timeline permitted.

In all countries, a series of *key informant interviews* was also carried out. Key informants were selected both at national and (to a lesser extent) sub-national level and included representatives of Ministries of Health, and of the HRH department within them, other Ministries or public agencies/bodies concerned with the health workforce (e.g. Health Service Board in Zimbabwe, Health Service Commissions in Sierra Leone and Uganda), health professionals associations, development partners, national and international NGOs, and faith-based organizations responsible for health service delivery (e.g. Zimbabwe Association of Church Related Hospitals).

The initial topic guide for the key informant interviews was the same for use across all of the countries. It was adapted to each of the contexts and also iteratively modified to reflect any emerging themes. The topic guide is summarised in Table 2. It comprised questions sequenced in chronological order and focusing on the HRH context before, during and (especially) after the conflict and the challenges faced in each of those periods. Secondly, the questions concerned the policy responses to those challenges, the processes through which they emerged and the effects they had for health workers as well as broadly for the health system. Interviews were kept semi-structured to allow the respondent to focus on those issues and policies, as well as time periods, of which they had more experience and knowledge.

**Table 2:** Summary of key informant interview guide

Examples of questions	
1.	Context and challenges
	• What was the situation of health workers after the conflict?
	• What were the main challenges in relation to the workforce, in particular with reference to recruitment, posting and retention in rural areas, motivation?
	• How did the challenges vary across the post-war period?
2.	Policy responses
	• Can you explain how the public policies changed over time since then?
	• What were the objectives of the new policies?
	• Did the policies build on what went before or not?
3.	Drivers of changes
	• What were the main factors which influenced the changes in policy?
	• Which factors do you think are most influential in policy change? (Please explain how and why). Specific people, specific organisations, funding, political factors, evidence, context changes (economic, security, political, organisational, international context..)?
	• Have these factors changed over the period? If so, describe how, and why.
	• Who were the main actors involved in the process of developing policies on HRH?
4.	Implementation challenges
	• Taking each of the major reform initiative in turn, can you describe to me how they were implemented?
	- What were the mechanisms?
	- Over what areas of the country?
	- Focussed on which health workers?
	- Implemented by whom?
	• What were the implementation challenges? Were they overcome? How?
5.	Financing & sustainability
	• How costly was the policy to implement?
	• Who funded it?
	• How sustainable do you think it is?
	• Is it still on-going? If not, why not?
6.	Effects of the policy changes
For each major policy change/intervention described, ask:	
	• Was it ever evaluated? How and by whom? What were the results?
	• What was its overall impact, in your view?
	• How did effects differ across regions? Across cadres? Across ethnic groups? Across genders?
	• How do others view the experience? What lessons have they drawn from it?
	• Did it have any unintended effects (positive or negative)?
More specific probes for impact on: health worker pay, recruitment, retention, distribution, performance, access to services and the health system more broadly	
7.	Your recommendations
	• Based on these experiences and what you described to us, which strategies you think should be adopted to address the current challenges for health workers?

### Data analysis

Data analysis was carried out at country level, separately for each of the tools adopted. The first, descriptive step of the analysis consisted in preparing a timeline which presents, in chronological order, the HRH related policies, reforms and practices which emerged during the period considered. Secondly, at a more analytical level, the qualitative information collected (e.g., documents, notes from stakeholder mappings and transcriptions of interviews) was coded either manually or using QSR NVivo 10 and ATLAS TI Version 7.0. In order to allow systematic comparison, the same or similar pre-identified themes were identified and used to code the information from the different sources and for all countries (Table 3). These codes are based on the questions highlighted in the study protocol [2].

**Table 3:** Themes used for coding of the information collected from the different sources

Themes	Subthemes	
HRH context and challenges	Recruitment challenges	Changes to these challenges over time before, during and after conflict/crisis
	Distribution challenges	
	Retention challenges	
	Performance challenges (pay, motivation, management, etc.)	
Policies adopted	Policy objectives and approaches	For each of the policy responses
	Drivers of change	
	Implementation of policies	
	Financing of policies	
	Impacts/effectiveness	

In this article, we compare and contrast the experiences, features and issues of the policy-making processes in each of the countries, using a policy analysis framework [20]. The Walt and Gilson policy triangle was selected as it is a relatively simple framework which includes all elements which were judged to be relevant to examine the evolution of HRH policies. Given the complexity of settings and time periods under consideration, a simple framework was appropriate to structure our examination of themes. Additionally, the systematic and deliberate comparison of multiple cases intends to improve the analytic generalizability of our findings by providing insights on the possible causal mechanism behind the policy-making patterns, thus allowing the development of more general and generalizable conclusions compared to single case studies [21, 22]. The issues on which the comparison focused, which are presented in this paper include (i) the HRH context and the challenges which emerged, persisted or were aggravated during and after the conflict/crisis, (ii) the policy content and the processes and patterns in the introduction and design of HRH policies, reforms and practices to address those HRH challenges over time, with a specific attention to the post-conflict/crisis phase, (iii) the drivers of the policy change, and (iv) the effectiveness of the policies and of their translation into practices.

### Study limitations

The methodology chosen has the advantage of providing rich data and information over the long time span which we aimed to cover in the case studies. In particular, the combination of methods within each case study allowed us to collect sufficient amount of information, in an iterative way, despite the general difficulties in gathering data for a long period of time and in post-conflict contexts where data are scarce [23], while the comparison of case studies allowed us to search for patterns and consider generalizability. Despite these advantages, the study has some limitations mostly due to the varying level of details between country cases. In some contexts, this was due to the difficulty of retrieving documents or for interviewees of recalling periods much before the data collection (e.g. in Sierra Leone). In other cases, this was due to the reticence of the interviewees, given the still fragile political situation (e.g. in Zimbabwe). Some elements of evidence – for example, on financing of policies – were harder to obtain. Finally, we focussed less on the probing for the *process* of policy making in this study (and more on the actors, factors, context, and content), although findings on process emerged indirectly from different sources.

### Ethics

Ethical approval for the research was obtained from the relevant national ethical committees in the four countries, as well as the Liverpool School of Tropical Medicine. Precautions were undertaken to obtain informed consent, to assure confidentiality of information, anonymity of respondents, to undertake research in a sensitive manner, and to keep data secure. Research locations were selected to ensure privacy and all data were anonymised.

## Results

### Context: challenges for HRH after the conflict

#### Recruitment

All four countries faced challenges recruiting adequate numbers of staff after the conflict or crisis, as would be expected, given that training institutions had been destroyed and large numbers of staff killed or fled. The most extreme example was Cambodia, where at the end of the Khmer Rouge regime in 1979, only 25 of the 450 medical doctors from before 1975 survived and remained in the country; and 26 pharmacists, 28 dentists, and 728 of the 3,400 medical students returned in 1979 [14]. This HRH crisis required immediate production of health professionals to reconstruct the health system. Similarly, in Sierra Leone and northern Uganda, the conflict depleted the number of health workers and exacerbated the main HRH challenges faced before the war. In Sierra Leone, in particular, health workers were targeted for abduction during the conflict to provide health services behind enemy lines [13] and many did not survive the conflict. In both countries, attrition rates for health workers also increased during this period, as many fled to safety with poor retention rates after the conflict, due to better economic situations elsewhere [8, 10]. In contrast, in Zimbabwe, given the different nature of the crisis, the challenge was more about retaining existing staff than recruiting new ones.

In addition, across all post-conflict countries, the production of health workers remained a challenge, as the in-country medical training institutions were not producing enough health workers to fill the gap created in the aftermath of the conflict. This compounded the problem of inadequate health workforce across all the cadres, though more pronounced for the higher cadres where starting numbers were lower. For example, only 67 medical officers were present in Sierra Leone in 2005 compared to 203 in 1993. The same pattern was seen for State Registered Nurses, of which only 152 of the 623 recorded in 1993 remained in 2005 [8].

Finally, recruitment challenges were made starker by a history of low, irregular remuneration for health professionals, which spans pre- and post-conflict. Many Zimbabwean staff migrated to the diaspora, while others simply absconded because there was no point in working due to inflation which rendered salaries worthless [24]. Structural factors, such as the recruitment ban and weak functionality of district service commissions were also identified as causes in Uganda [25].

#### Distribution

Across the four settings, distribution of staff was a major challenge – one which had pre-dated the conflict or crisis, but was worsened by it. In Uganda, for example, the Acholi sub-region in the north of the country had a poor HRH status compared to other regions, with the majority of districts in Acholi being below nationally-defined staffing norms [11]. Key informant interviews also highlighted the challenges of poor staff mix, unbalanced gender mix and absence of key health staff required at various levels of health facilities [25]. The situation was worsened by the absorption of clinical staff into administrative roles, as one of the key informants in Uganda recounted:

*“We [now] have one doctor who is the DHO [district health officer] … of course he is an administrator… Other cadres like nursing officers, midwives are still lacking. We have [only] filled around 46 % of the staff [required] … Such is the dilemma we are in.” (KII - Amuru, Uganda)*

In Sierra Leone, health workers that remained after the conflict preferred to work in the district headquarter towns, leaving rural areas grossly understaffed [26]. Similarly, prior to 1995, the challenge facing the Ministry of Health (MoH) in Cambodia was not only to quickly produce health workers, but also to ensure their equitable distribution from Phnom Penh and its surrounding provinces to other secured districts as well as to address the unbalanced distribution of health facilities [17]. Zimbabwe presented a slightly more complex picture – while the loss of health workers affected the rural areas disproportionally, especially in terms of higher level staff, interviews pointed to the fact that some staff preferred to be posted to rural areas during the crisis in order to reduce living costs [27].

#### Retention

Pre-existing challenges relating to the retention of health workers continued and were aggravated in the aftermath of the conflict in some settings. Low and irregular payments, lack of promotion and clear career progression, unavailability of suitable accommodation and generally poor working conditions, all contributed to retention challenges in the public sector in Uganda, for example. In particular, the low-level and delayed payments were found to be caused by financial limitations and wage bill ceilings [11] – a finding across all four settings and in common with many other low income countries.

Low financial remuneration for health cadres meant that working for NGOs tended to be far more attractive to health workers than working in government health facilities, due to the better financial incentive and training environment, as reported in the three post-conflict countries.

*“It was horrible. The health personnel had migrated outside or were working for NGOs. There were critical shortages.” (KII– MoH, Sierra Leone)*

With the end of conflict, the departure of NGOs led to the inability to sustain staff whose salaries were previously paid by NGOs. In northern Uganda, this meant that many health workers moved to neighbouring South Sudan, which was then (at the time of data collection) experiencing a post-conflict aid boom and thus attracted NGOs paying better salaries and providing better working conditions.

*“The majority of our staff are going to South Sudan because these days there are few NGOs but in Sudan, they are better paid than in Uganda.” (KII - Kitgum, Uganda)*

These HRH movement patterns highlight the interconnection of health labour forces within the region. Another pattern can be found in the movement between sectors. In Uganda, there is a reported increase in movement of health workers from private non-for-profit sector (PNFP) to public sector in the post-conflict period, mainly due to health workers’ experiences of the PNFP (largely mission) sector, better pay in the public sector and other retirement-related incentives [28]. Similarly, in Zimbabwe, the economic crisis has generated inter-sectoral movements of staff, with staff seeking employment by municipalities, which are able to offer more attractive incentives while regular salaries lost much of their value in the crisis. This has led to perverse outcomes, with more experienced staff at lower level facilities which are able to offer better overall pay [24].

In Cambodia, retention did not emerge as a major challenge in the initial post-conflict phase, at least for the secure areas of the country. Despite poor road conditions and a lack of infrastructure, health workers were reportedly more than willing to stay and work in their assigned posts. Getting government employment status, social recognition and a sense of helping people were cited as motivating factors to remain working. In addition, before the mid-1990s, there was little difference in living conditions between urban and rural areas, and the local recruitment system worked well particularly when people were posted to their home areas [17]. Shortages of well-trained health workers prior to 1993 meant that doctors and other well-trained cadres were often placed in the provincial towns, rather than working in rural areas. After the national election in 1993, health managers reported greater challenges in posting and retaining health workers in rural areas. Insecurity was then replaced by a more complicated set of issues for retention, including social and economic opportunities for staff. For example, the growing private sector offered dual practice opportunities in urban areas. Additionally, the influx of external actors to support the reconstruction and development of the health sector led to a brain drain from public to NGO sector or development programmes. Since 2002, however, turnover has been relatively low in the public health sector, according to official records. Staff attrition is estimated at around 1-2 % per year across the entire workforce and around 4 % for primary nurses in the public health sector.

#### Performance

##### Workload

The difficulty of re-staffing the sector and retaining staff had knock-on effects on workload in all settings, though in some, like Cambodia, this has been less marked because of low demand and access to health services, at least in the initial post-conflict period. Health workers in northern Uganda lamented that, with the closure of the refugee/IDP camps and the return of the population to their villages, the workload increased because of the difficulties in mobilizing and reaching patients within a larger catchment area,

*“… [In] camps … everybody would conveniently come for treatment but now everybody has gone back to their villages some distance away and so health workers have to commute to those places, which is really challenging.” (KII - Kitgum, Uganda)*

##### Motivation

Across the countries, health workers reported low motivation due to low salary levels, lack of career progression, delayed salaries (in some cases such as Uganda, caused by un-updated payroll), and insufficient or absent accommodation for staff at health facilities [13, 25]. Some aspects, such as housing, have received investment in post-conflict recovery plans, whereas others, like career progression and difficult working conditions, persist. Our analysis also revealed that the issue of low pay levels was not cited as a challenge in the immediate post-conflict period, although remuneration increased in importance for many staff over time as systems were re-established and expectations grew. In Zimbabwe, however, given that the crisis was dominated by an economic collapse, pay was the core issue - the most conspicuous change that occurred was the marked decline in the standard of living. Depressed salaries and dissatisfaction with working conditions became widespread and from 2004, the vast majority of health workers were beginning to do extramural economic activities to be able to sustain themselves. In particular, the practice of selling a variety of wares at the workplace was very prevalent during the crisis in all the districts and healthcare sectors studied [27].

##### HRH coordination

In the immediate post-conflict period in Sierra Leone, it was reported that a number of different actors were involved in the health system reconstruction. However a lack of coordination between these actors, described as chaotic, lead to a fragmented approach to reconstruction, which posed a huge challenge for the MOHS to establish control over the health workforce [29].

*“After the war, it was complete chaos. The NGOs came and went […]. They employed the nurses directly, without even consulting the Ministry. […] They never presented any budget. But this was a war. We had to bend backwards in the Ministry” (SM – MoH, Sierra Leone).*

The pattern does vary by country however. In Cambodia, this proliferation of players was also found, though not immediately post-conflict, while in Zimbabwe, given the strained relationships at national level, the influx of aid players was more limited. In Uganda, given the protracted nature of the conflict, NGOs and donors were present and supporting work in northern Uganda from the crisis period; the post-crisis period has seen the continued presence of some with some degree of withdrawal now too.

### Content: policy responses

#### Uganda

**Fig. 1:**
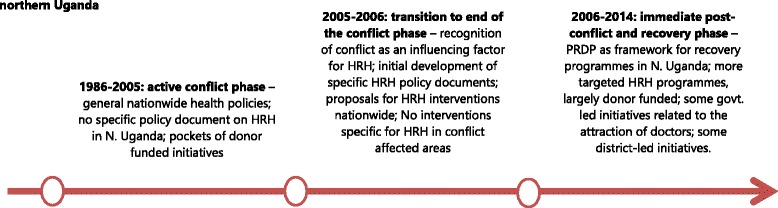
Evolution of HRH policy responses in post-conflict Northern Uganda

While sharing many of the challenges of the other settings, Uganda’s policy responses show a specific pattern, which in large part reflects the fact that in this case study, conflict had affected only one region of the country. The rest of Uganda had a settled policy-making system, which continued to provide overall guidance, with limited recognition of the need for specific regional responses or, initially, specific planning for HRH as a sub-sector (Fig. 1). From 1999 to 2005, there were no policies on HRH, rather HRH issues and challenges were merely indicated as sub-sections of broader nationwide health policies. Generally, responses to HRH challenges were nationwide, with no particular focus on conflict-affected areas in Uganda [11, 25].

In late 2005, the second *Health Sector Strategic Plan* (HSSP II 2005/6-2009/10) included for the first time the recognition of conflict as a factor influencing the health sector and human resources (Namakula et al. 2014). The following year, the *Human Resources for Health Policy* (2006) and the *HRH Strategic Plan* were developed to propose policies to address the identified challenges. This included a motivation and retention strategy, as well as a hard-to-reach policy (2010) with the aim of ensuring the retention of health workers in hard-to-reach areas, and particularly those affected by the conflict [11]. These documents were prepared in the context of a general drive to introduce specific policy responses and interventions in post-conflict northern Uganda. In terms of HRH, other strategies in place focused on improving recruitment, working conditions, retention of health staff, particularly midwives and medical officers, as well as addressing their training needs. All of these policy responses were implemented under an overarching framework called the *Peace Recovery and Development Plan* (PRDP 2007). With the exception of a few district-led initiatives, the majority of these initiatives were donor-funded [11, 25]. In contrast, national initiatives have been discussed but rarely granted sufficient resources to guarantee implementation. Moreover, periodic recruitment bans were introduced which hindered health worker recruitment in the public sector. As a consequence, most initiatives on the ground to improve staffing, such as scholarships and support to in-service training, remained tied to donor-funded projects [11].

#### Sierra Leone

**Fig. 2:**
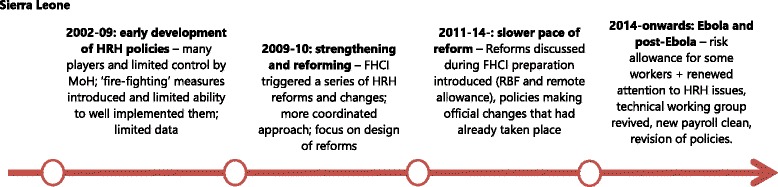
Evolution of HRH policies in post-conflict Sierra Leone

The development of HRH policy making in post-conflict Sierra Leone is presented in Fig. 2 (see also [8]).

The first phase, in the immediate post-conflict period (i.e. 2002–9), consisted mostly in what one of the key informants referred to as ‘fire-fighting’. This phase was characterized by the presence of different actors involved in the health system reconstruction. However, a lack of coordination between these actors led to a fragmented approach to reconstruction.

*“People started working on their own areas and they started developing a policy and plan and things like that […]. But it was all happening in parallel, also depending […] on the focus of donors to provide TA [technical assistance] and funding for certain things. So I think a lot of policies applied at the beginning were definitely donor-driven. WHO said ‘you don’t have a policy on this and this. We have to develop it’, and you’ll get it.” (KII – NGO, Sierra Leone).*

The catalyst for change or the ‘window of opportunity’ for effective HRH reform was the launch of the Free Health Care Initiative (FHCI), which was announced in 2009 [30] in response to poor maternal and child mortality rates. A series of reforms was introduced to effectively operationalize the FHCI, and HRH issues took a key place among these. These included a significant salary uplift for technical health workers, fast-track recruitment of health workers at district level, updating the payroll to reflect those already volunteering in the facilities, and a Staff Sanction Framework to monitor absenteeism and protect the investment of the FHCI in 2010. In 2011 and 2012, a Performance Based Financing (PBF) scheme and a Remote Area Allowance were introduced to provide motivation for primary care workers and remote staff. However, by 2012, the reforming momentum that drove the design of these reforms was lost, and their implementation lagged behind and faced numerous challenges [30, 31], entering a new phase of slow paced policy-making. In this phase, the new *HRH Policy* and *HRH Strategic Plan* (2012) were prepared, giving *ex-post* shape to the changes that had already taken place at operational level [8].

The 2014 Ebola outbreak led to a collapse in the already fragile health system, with disastrous consequences for communities and staff. After a delayed response, emergency measures were implemented, including support and risk allowances for health workers in 2015. As control of the outbreak improved and the country moved to the post-Ebola health system strengthening phase, there is renewed interest in HRH. The Technical Working Group initially created during the preparation of the FHCI has been revived in 2015 and charged with the coordination and implementation of HRH strategies and activities. Initially, these included another payroll clean and a revision of the 2012 HRH policies and strategies [31].

#### Zimbabwe

**Fig. 3:**
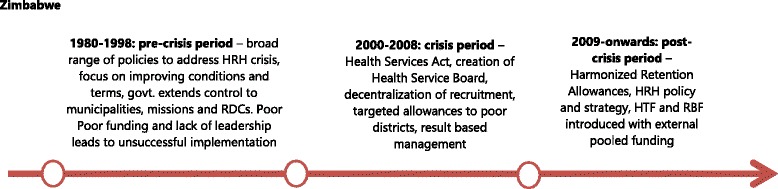
Evolution of HRH policies in pre- and post-crisis Zimbabwe

Figure 3 shows the main phases of HRH reforms before, during and after the crisis in Zimbabwe. After independence in 1980, in the period which predated the crisis, the main focus for the health sector in Zimbabwe had been on extending coverage of services. In the 1990s, in terms of HRH reforms, three key policies were introduced: (i) the job evaluation exercise (1992), (ii) the adoption of the performance appraisal system (1996), and (iii) the unification of the various sectors by government (1997) to ensure better coordination [24]. The job evaluation exercise had initially found that salaries for those working in the public health sector were low compared to personnel in state enterprises and private sector, and the government accepted in principle to progressively increase salaries of HRH over a three year period. This decision, however, was never implemented. In 1996, a public service-wide performance management appraisal system was introduced to ensure that high performers were rewarded accordingly and hence would be retained. However, the implementation of the scheme was poor because of low capacity to manage the system and lack of transparency, so that the system was turned into a mechanism for settling scores, as well as because of lack of funds to support the salary awards. Finally, the 1997 takeover of management of health staff in the PNFP (largely faith-based) sector by the government was meant to achieve greater equity in service delivery as well as to ensure uniform HRH terms and conditions. However, the reform did not work fully, as service contracts between government and mission providers were not implemented in some provinces [24, 27].

The crisis started in 2002. During this period, policy-making concerning HRH continued with key reforms such as the promulgation of the Health Service Act (2004), which paved the way for the formation of the Health Service Board (HSB), in an effort to better respond to HRH pay and management needs. In 2005, a performance appraisal and management system, called Results Based Management, was adopted. In 2007, several reforms were adopted, including hospital boards (tasked with the recruitment of selected health worker grades), duty free importation of vehicles for certain health worker grades and a targeted incentive scheme in the 24 poorest districts. However, as in the previous period, all these reforms had limited support, funding and implementation [27].

The situation changed in the post-crisis period. At that time, initiatives which were introduced to address the on-going HRH challenges, such as the harmonised HRH retention allowance (2009), a Results Based Financing (RBF) scheme (2011), and the Health Transition Fund (HTF) in 2012, enjoyed the support of the pooled donor funding which was created following the re-establishment of the relationships with donors. They were more actively and effectively implemented compared to the policies in the previous periods.

#### Cambodia

**Fig. 4:**
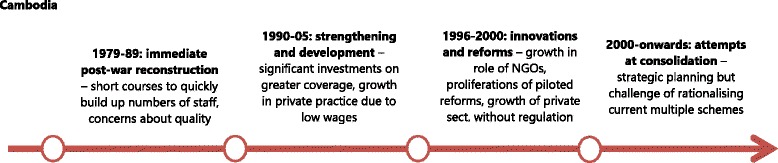
Evolution of HRH policies in post-conflict Cambodia

Cambodia, with its longer post-conflict time-span, has gone through a number of main phases of HRH policy evolution (Fig. 4). During the initial reconstruction phase (1979 to 1989), the focus was on rapidly increasing production of staff, given the very low starting numbers. This was attempted by restoring the training facilities at national, regional and provincial levels [6]. However, the quality of health workers trained varied because of uncoordinated and outmoded training curricula and lack of expert trainers. Additionally, with a view to cascading skills, students were selected by the provincial health departments (PHDs) and then were deployed to their districts and/or commune of origin. The health mangers recalled that:

“[…] *we used a slogan: ‘the one with more knowledge trained the one with no knowledge’. Regardless of quality of trainings we have, but this way of knowledge transfer at least worked well to have one or two few trained staffs at the commune clinic or health post at that time.”* (KII – Manager, Cambodia).

During a second policy-making phase, between 1990 and 1995, after the first national elections of 1993, Cambodia began to receive an influx of official development assistance to rebuild, including the health sector. This led to an increased presence of international NGOs, which grew from 23 in 1988 to 164 in the mid-1990s [16]. The MoH, with technical support from WHO, developed health policies, plans and institutional mechanisms to coordinate external assistance [32]. However, it was constrained by limited managerial capacity, shortages of health workers and low levels of government funding. Meanwhile, the conflict between government and remnants of the Khmer Rouge continued until 1997 and many districts could not be accessed. Coverage and functionality of facilities in those districts remained extremely low. In this period, the MoH maintained the focus on strengthening training institutions. Key informants reported that the emphasis was still on boosting numbers and filling gaps, and that quality remained low [17].

In the next half decade (1996–1999), a number of health management innovations were introduced, including the first Health Coverage Plan (HCP) in 1995, the Health Financing Charter (HFC) in 1996, and the introduction of health operational districts (ODs) in 1997. Most of these were not focussed on HRH as such but had major implications for health staff. For example, in 1996, the government endorsed the collection of user fees at public facilities in order to reduce under-the-table charges and increase utilisation [33]. The HFC aimed to strengthen the professional code of conduct of health staff and increase revenue for public facilities, which was then redistributed among the health workers. In 1998, the government allowed international NGOs to pilot a number of financial incentives schemes, including Community-Based-Health Insurance (CBHI) in 1998 and contracting in and out of health management at the OD level. All of these changed the working conditions and incentives for staff. The government had taken advantage of the NGO managed schemes to deploy key health professionals in rural areas. With regard to HRH production, the secondary midwife course ended in 1996 with no training of new (primary and secondary) midwives until 2003. This was in response to the change in focus, mostly driven by development partners, from quantity to quality of human resources development. At the same time, the MoH rationalised 59 categories of health workers into 29 equivalents [6], and pre-service education for physician assistants was terminated.

In the final phase documented, from 2000 to the present, the government has been learning from the previous periods of experimentation to scale up initiatives which were positively assessed, with the aim of increasing coverage but also national ownership. In the first HSP (2003–2007), human resource development objectives included increasing the number of midwives through basic training and strengthening the skills of midwives already trained through continuing education; addressing the mal-distribution of key health personnel and improving the retention of well-trained health workers, especially in rural areas. The second HSP (2008–2015), which was accompanied by a health workforce development plan, focused on aligning human resource planning and personal management with heath sector planning, developing and implementing HRH management policies to deploy staff in underserved areas through contracts, and increasing the number of midwives placed and retained in public sector facilities through effective financial incentives.

These measures included reform of civil service remuneration, under which base pay increased by 10 % in 2007/8 and then 20 % per year for the next few years (World Bank, 2013). Alongside this, an array of salary supplementations were introduced or continued, including from user fees, the health equity funds (introduced in 2000), service delivery grants, contracting (operating in various forms since 1999), special operation agency (SOA) funds (a form of internal public sector contracting, starting in 2003), and community based health insurance (CBHI). In addition, in 2008, a government midwife incentive scheme (GMIS) was introduced, paying between USD 10 in urban areas and USD 15 in rural areas for every live birth, alongside vouchers to cover demand-side costs (transport and food allowance for the poor) from 2010. These reforms focused on boosting the level of resources available, effective management and incentives to improve staff commitment to increase the quantity and quality of service delivery. Among these financial innovations, the demand-side schemes (CBHI, vouchers, HEF) are still managed and implemented by the international NGOs. Only the user fee formalisation and GMIS are operating on a nationwide scale, though other programmes are being scaled up gradually. There is a plan to develop social health insurance out of the CBHI experiences, but this is not yet agreed [34].

### Actors and factors: drivers of change

Our interviews and document review explored the actors and factors behind the changes observed in HRH policies. The themes which emerged included the role of the development partners, economic factors, political changes (at home and internationally) and the role of evidence and advocacy. We look here for patterns across the different contexts.

#### The role of the development partners

It is not surprising that in these fragile and post-conflict settings, development partners were found to play an important role, though there is considerable variation across different phases and places and in the kinds of roles played.

In Uganda, where a stable polity had continued in the south during conflict in the north, the development partners’ role seems to have mainly been exerted through funding of different elements of the reconstruction plan and supporting NGO-led projects, rather than technical assistance to national policies and plans. The end of the conflict enabled the government to regain a lead coordination role over other actors:

*“I think there is more order now that the conflict has ended, I think the government is going to become much more influential and I think already planned initiatives are going to be implemented according to plan, rather than people just coming in to address an emergency in which case they could justify any thing. They could justify their presence in so many ways because people are dying, children are sick and everyone is running around looking for a solution. So I think post-conflict there is much more systematic way of implementing things.” (KII - Kampala, Uganda)*

However, the availability of funding remains a key factor in policies and programmes being implemented, rather than remaining on paper only.

*“[…] you can approve but if there is no money then you can’t do anything. They can say ‘okay the policy is good but we can’t implement it this year because there is no money to include in the budget’; then there is nothing you can do.” (KII- Kampala, Uganda)*

The recession led to a remarkable reduction in donor funding, particularly for the PNFP sector which relied on this for e.g. the payment of staff salaries in Uganda.

*“The hospital has really faced a lot of challenges because of the economic changes which are happening. So in a way, last year, so many donors withdrew and the hospital budget was greatly affected. It was so difficult for the hospital to maintain the number of staff which led to reduction in their number. There was restructuring.”(KII – Kitgum, Uganda)*

In Zimbabwe, the economic crisis led to development partners taking a lead role in sustaining the health workforce, at least for a period, though there is less evidence of a role in influencing policies, presumably partly because of the higher capacity of the Zimbabwe system prior to collapse as well as the tenser government/donor relationship.

*“Normally Global Fund does not support salaries in any way so it was a special request by the Ministry to Global Fund or even to the HTF [pooled donor funding] now that they support so that if people deliver, their salaries will be provided for and they agreed in the hope that the government will improve salaries and they are hoping they will be able to support its workers”* (KII - National, Zimbabwe)

*“… during the crisis particularly in 2007 when the health sector was almost collapsing these development partners influenced government to adopt policies that sustained HRH supply in some of the most disadvantaged provinces and districts. Government allowed health workers to be paid directly by development partners in foreign currency in selected districts”* (KII - Development partner, Zimbabwe)

From 2000, international development partners and bilateral and multi-lateral players lost confidence in the government and gradually began to reduce financial support to the health sector. Instead of direct support to government, donors opted to channel financial support through implementing partners and other pooled funding mechanisms. An example was the Vital Health Services Support Programme, funded by the European Union and the Global Fund, which was instituted in September 2007 in three districts in each of the eight provinces that paid incentives through the district health fund.

The political settlement in 2009 brought with it a sense of economic stability and international cooperation. The coalition government facilitated cordial relations with development partners which saw the injection of funding towards re-attracting health workers to health facilities in rural areas. Key informants noted that the introduction of the donor-supported harmonised retention allowance in March 2009 marked the beginning of a process to improve the distribution of health workers. The normalisation of staffing levels was a major achievement of the coalition government which happened against the back-drop of improved funding streams from the international donor community. However, key informants noted that donors continued to mistrust government. The harmonised retention fund was being managed by Crown Agents, for example, and participants said this was done to improve accountability.

In Cambodia and Sierra Leone, development partners appear to have had more influence through technical assistance as well as financing. Many of the HRH policy documents were produced by and to some extent for international agencies in the first post-conflict period in Sierra Leone, while in Cambodia, some important shifts, for example, from quantity of HRH production to quality in the 1990s were reported to be driven by development partners.

The expectation of a period of intense dependence on development partners post-conflict, followed by a smooth transition to government leadership, is not supported by all case studies. In Cambodia, for example, development partners have been very influential over policies and programmes for a long period, with dependence if anything intensifying in later phases as multiple innovations were developed, piloted and evaluated by a combination of external funders and NGOs. In Cambodia, in the 1990s, the priorities of capacity building, HRH development and management were pulled into different directions by conflicting agendas of multilateral and international agencies and lack of coordination. This situation led to overlapping interventions and did not help strengthening government stewardship and ownership of health sector development in the post-conflict period. However, by the 2000s, a more coordinated approach from both donor and government side emerged, including through a sector-wide approach [17].

In Cambodia, as in other settings, formal procedure may be relatively less important in decision-making processes compared to behind-the-scene negotiations [35]. MoH staff with good technical knowledge holding powerful positions provide an asset for developing policies and strategies because they are required to deal with donors and technical assistant expatriates. However, the donors are influential in shaping policy making and priority actions because of the significant amount of funds they had for health sector. Among those donors, WHO has played a leading role in providing technical support to the MoH for policy formulation since 1993 [6].

In Sierra Leone, the periodic crises linked to conflict and Ebola have led to a cycle of higher cooperation and dependence on external organisations. In the post-conflict phase, challenges were reported for the government to assert effective leadership in the health sector and for HRH [8, 36]. In Zimbabwe, the unresolved political and economic issues perpetuate the importance of development partner contributions to maintaining staff, even as donors are excluded from influence in other respects [27].

#### Implementation partners and NGOs

While development partners seem to have played a key role in defining policy making at central level, at local level NGOs and non-public actors were critical to shape the implementation of policies and practices, and in many cases ensure service provision.

In Uganda, although the government remained the lead provider of health services, during the conflict period NGOs and PNFPs took an increased role. After the conflict, many of the NGOs closed the projects or scaled down their operations in northern Uganda, leaving the local and national government to assume a lead role as before the onset of the conflict. This early departure may be due to the long-term nature of the conflict in northern Uganda (which meant a presence in the districts during the conflict by NGOs). In Cambodia, by contrast, the role of NGOs as implementing agencies grew over the post-conflict period, spurred on by the total destruction of the health system during the war, the initial fragmentation and dependence of the government on external support and a consequent openness to innovation. In Sierra Leone too, NGOs entered post-conflict and have retained an important operational role, not least because trust in public finance systems remains weak. In the post-Ebola period and post-conflict, donors prefer to channel a part of their funding through NGOs. Analysis of health worker incentives at district level found that NGOs played an important role by influencing pay and activities through the material and other support provided for different programmes [37].

#### Political leadership

Although external actors have played a key role in defining policy-making patterns in post-conflict environments, political leadership can also be, in some contexts, a critical factor. In Sierra Leone, for example, political leadership was central for the development and sustaining of the FHCI [30, 31]. As with most major policy developments, a constellation of factors was identified which supported this move, including the influence of the international context (favourable to removal of user fees at that time and promoting the MDGs) and the support of external players like the British Government and other donors. In addition, the context of poor maternal and child health indicators and evidence of the role of financial barriers in preventing access were important underlying factors.

“*It was a presidential initiative and so people were interested, that’s one. Number two, there’s been a cry for attention towards maternal and child mortality and for many, many years Sierra Leone was last in the human development index”* (KII - MoH, Sierra Leone).

#### Ministry of Health

The influence of the Ministry of Health over HRH policy-making does not emerge clearly from interviews, which is surprising but may reflect a combination of institutional weakness post-conflict with the twin pressures of strong donor influence and political centralisation.

In Cambodia, the drive to regain control and ownership over HRH and management processes by the Ministry of Health as distance in time from conflict is reached is evident in a number of different areas, but best illustrated by the story of contracting. In 2009, the government adopted a contracting-in model called SOAs, which built on lessons from previous models - ‘contacting-in and out’ of health management in five ODs in 1999 – 2003, and hybrid contracting piloted in 11 ODs between 2003 and 2008. While these earlier models had been evaluated as successful in some respects, they were seen as costly and too dependent on external actors. The MoH’s efforts to bring control back into the public sphere led to the SOA model, based on performance contracts within the public sector.

#### Other public actors

HRH policies can clearly impact on other sectors and the public purse, making collaboration with Ministries of Finance and other public bodies important to the making and implementing of policies. In Zimbabwe, for example, many of the policies introduced to allow for improvements to HRH terms and conditions (in order to improve retention during difficult times) were stymied by an unwillingness to allow the health sector to be different from other civil service sectors.

*“The government service is not divisible and no single department or ministry can decide to be treated differently; if you study the public service regulations that is made very clear so the health service will always be part of the civil service and the PSC will monitor the MOHCW” (*KII – manager, Zimbabwe).

#### Political and economic changes

The end of conflict and the establishment of a stable government are generally prerequisites for policy making and smooth policy implementation. In Zimbabwe, after a period of political chaos, the formation of the new coalition government as a result of the Global Political Agreement brokered by the Southern African Development Community on 15 September 2008 brought about a semblance of normalcy and this began to restore confidence in the health sector.

*“The unity government changed a lot of things and cadres in the health sector began to feel confident and the introduction of the United States dollar improved the economic condition of health workers. As you know most of the health workers were in the country doing whatever could sustain them. The worst thing that happened during the crisis is that cadres could not access their salaries so no one could blame them for not coming to work because in reality these cadres were working for nothing”* (KII -manager, Zimbabwe).

Clearly the economic situation in Zimbabwe was not only a key driver of the crisis but also shaped the policy responses to it, including the unwillingness and inability to adequately fund the sector, and hence the dependence on development partners, noted above. The macroeconomic situation in Zimbabwe deteriorated at an unprecedented rate and to severe levels between 2007 and 2008. During this economic meltdown a sharp increase in health worker migration from the country occurred. The exodus reached a level where the public health sector, according to one key informant, *“became so dislocated that it was difficult to refer to it as a health service at all in some areas of the country”* [27]*.* Economic trends also affected income-generation at lower levels (e.g. in municipalities) which directly influenced the package of incentives which could be offered to health staff.

International politics are also a major driver of policy change, which affects all areas including HRH. For example, Cambodia was subjected to international sanctions until 1991 and had to rely in that period on humanitarian assistance from some socialist countries and a few international NGOs [15]. Following the UN-sponsored national election in 1993, the influx of external development agencies and funds could begin.

#### Evidence

The role of evidence in driving HRH policy making must also be considered and there were examples of evidence feeding back into policy in all four countries. However, it is not always clear the extent to which evidence drives policy or is used to support already-made decisions.

*“The Presidential Review Commission of 1999 led to the formulation of important policy agendas; the HSB was born out of the findings of the commission. […] Assessments and situational analyses have led to changes in policy indicating that evidence can sometimes be a factor in policy change. The 2008 HRH situational analysis informed the formulation of the harmonised retention allowance policy.”* (KII - manager, Zimbabwe)

*“Before [the FHCI] we actually did a survey together with [another NGO] and our finding was that money was a barrier for women and children to access the health service delivery. So we started a case to the government, together with other INGOs, for user fees to be removed for the care of women and children.”* (KII - NGO, Sierra Leone)

The country which has benefited from the most intense operational research is Cambodia, where changes in policy seem at least in part derived from evidence feedback loops, maybe because of the more operational presence of NGOs and long term engagement by development partners.

#### Advocacy

Advocacy carried out by groups and institutions (e.g. workers unions, parliamentary committees, NGOs) working in the health sector emerged as a driver of change in health policy in Uganda. The policy on hard to reach allowances for health workers working in remote districts was one such policy for which workers unions had campaigned. The role of effective local lobby groups may be a marker of a settled polity, which may explain why this factor was only mentioned in the Ugandan interviews.

*“What we have been having is that the parliament has lately been involved in these issues […]. The parliament is more defined in terms of research, budget and more technical people and the members are more concerned. This led to strong support to health-related agenda but also the other point is we have been having dynamic workers association which is Uganda nurses’ association. Uganda workers union and other associations and union have become more organized and serious and then we also have the civil society organizations that have been very active.” (KII - Kampala, Uganda)*

### Policy effectiveness

In this section, we are not concerned with assessing the effectiveness of specific initiatives in each country so much as drawing a wider conclusion about the effectiveness of the policy trajectory in these post-conflict settings – the extent to which it matched to needs, addressed them and was informed by learning processes (i.e., an understanding of what worked or what did not, why and how to address critical blockages).

In Uganda, health policies like the Health Sector Strategic Plans (HSSPs I,II and III), the PRDP 2007 and 2010, which is the overarching policy framework for recovery, and the five-year donor funded implementation programmes had either mid-term or annual evaluations embedded [11]. These policies had an impact on the general working conditions of health workers but were mainly focused on health worker numbers as the main metric of success. Most other policy initiatives such as the hard to reach allowance (2010) and bursary scheme (2009) have not been evaluated. In general, KIIs indicated that there are pockets of improvements as a result of policies, but these are still far from the planned targets, both at national level and those desired by health workers in conflict-affected areas [25]. Hence, the HRH challenges identified in the early post-conflict period have persisted. The effectiveness of the policies has been hindered by limited funding, limited capacity of some actors, poor coordination, and lack of support and supervision, among other factors [11].

In Sierra Leone, the series of reforms that accompanied the launch of the FHCI have been assessed as relatively successful in terms of addressing the most pressing HRH issues [30]. These reforms contributed substantially to the rationalization and improvement of the incentive package available for health workers. However, it is interesting to note that most of the respondents, especially those working at central level, focused their narratives almost exclusively on the design and the planning phase of the reforms. Few of them discussed the implementation phase and the challenges it brought, or were aware of any evaluations of the impact of those reforms. This suggests that attention was given to the design of the policies and far less focus was applied to the implementation and how policies were translated into practice. For example, there was very little awareness at central level about the PBF and remote allowance schemes – both critical to health worker motivation and facing considerable implementation challenges. Additionally, the few evaluations of policies were externally commissioned (for example, the FHCI one [31]).

During the launch of the FHCI, preferences were also given to one-off exercises, e.g. the mobile recruitment programme, or shorter-term solutions, rather than an organic and coherent reform package (such as addressing pre-service training) [38].

*“On the package for reward, incentives […] it was a bit lost, not looking at the international evidence. […]. I don’t know how you would say that, but a kind of bricolage”* (KII – donor, Sierra Leone).

Despite the increase in the alignment of partners with ministerial policies at least at design stage, the lack of coordination became problematic after the launch of the FHCI, when the political pressure for rapid reforms was reduced, leaving room for fragmented policy-making and implementation. Disconnections appeared between MoH and donors, among donors, and even between the different departments of the MoH and at different levels of the administrative hierarchy (central and district-level). The result was a series of policies that were not completely coherent and a largely ineffective implementation of these policies. Additionally, the dependence on donor funding and technical assistance has so far led to a cyclical pattern – windows of opportunity (generated by crises or political leadership, backed by external support) which bring funding and focus, followed by fallow periods of stagnation. This points to underlying institutional weaknesses as well as domestic financing and capacity constraints [36].

In Zimbabwe, policy developments like the creation of the Health Service Board to implement initiatives that addressed health worker needs were ultimately not able to provide an acceptable package across all of the sub-sectors and there was little impact on recruitment and retention [24]. In the absence of higher level political will and financing, shifting institutions cannot address the fundamental blockages, even though the understanding of the problem by all major stakeholders was clear. The only initiatives which have shown palpable results have been coordinated by donors, such as the introduction of the harmonised retention scheme in 2009, which led to improved recruitment of nurses, doctors and environmental health professionals [27].

In Cambodia, the number of health workers employed within the MoH system has significantly increased over time, especially midwives posted in rural areas, though challenges still remain to reach planned numbers of other key health personnel and the health information system for health staff is not fully reliable [39]. Little progress has been made in addressing the maldistribution of health workers between urban and rural areas or in improving working and living conditions with appropriate incentives to attract and retain well-trained health workers in underserved areas of the country. KII also expressed concerns about the clinical competency of new midwives and nurses placed in rural areas, given the limited capacity and resources of the accrediting body, and the lack of systematic in-service training for health staff in rural areas. The recentralisation of recruitment in 2002 was intended to address quality concerns but appears to have exacerbated the difficulty of retaining staff in remote areas. The MoH was reported to achieve only 50 % of its annual personnel recruitment plans in some years, leading to the hiring of local staff on temporary contracts to fill gaps [17].

The policy process has demonstrated a growing confidence on the part of the MoH and a growing harmonisation amongst donors, as well as a willingness to learn from evidence while meeting the needs of the moment, which has led to higher priority being given to different policy objectives at each stage. Some underlying issues remain unresolved, however, including a remuneration package which is not adequate for health staff, and in particular for those in rural areas without substantial dual practice opportunities, and which fails to incentise public service adequately. In addition, the financial incentive schemes are complex and time-consuming to administer at the local level [17].

## Discussion

Our findings on the HRH challenges faced in these four setting are consistent with one another and with the wider literature [5, 40, 41]. As a result of conflict and crisis, the number of health workers diminishes because of death and migration and additionally health workers are likely to move to the more secure or economically stable areas, therefore increasing the unbalanced distribution within the country. The skills and quality of health workers worsen, because of the lack of in-service training and supportive supervision, but also because of the expansion of below-standard training institutions. Their productivity also decreases because of absenteeism, poor working conditions, unavailability of drugs and equipment, low salaries and demotivation. Often, given the weaknesses or absence of regulation and control, health workers put in place a series of financial coping strategies which further weaken their performance and the health system, including dual practice, both in the health sector (private practice or work for NGOs and aid agencies) and outside [40].

The main differences between challenges and responses in these four settings were driven by the length and nature of the conflict or crisis, whether it was resolved effectively or lingered, and how long its aftermath lasted. In Uganda, conflict was long but only affected one part of the country, and is now resolved, at least for the moment. In Sierra Leone, the conflict was total but resolved with international assistance, although the country faces on-going fragility made worse by the consequences of the Ebola outbreak. Zimbabwe remains in a state of chronic political and economic crisis. Cambodia faced total collapse and a prolonged period of partial peace, but is now stabilised and moving beyond ‘post-conflict’, at least in the eyes of most research participants.

Our findings suggest that there is no formula for whether or when a ‘window of opportunity’ will arise which allows health systems to be reset or to break free from path dependencies defined by the choices made for the previous system. In Sierra Leone, there was a moment of reform in 2009–10 with the FHCI, but this was eight years post-conflict and related more to a constellation of political will and coordinated donor support than the earlier crisis. The current post-Ebola transition appears to be another moment of opportunity for the country due to the influx of funds, development partners and NGOs. However, in the opportunity is also a risk of loss of coordination as the capacity to manage is often lowest at these crisis moments.

There is no evidence of a moment of seismic change in human resources for health policies in the other three settings, where path dependencies are more evident. In Cambodia, the early choice of contracting out service delivery has created a legacy of long-term experimentation and fragmentation of HRH management and remuneration approaches, which the government is now looking to harmonise. In Uganda, national policies and policy processes have been extended to the north, with limited concessions to its different post-conflict needs. In Zimbabwe, political and economic stasis has prevented known HRH challenges from being resolved, except by stop-gap external programmes. This pattern may in part relate to the nature of the post-conflict or crisis political settlement: in Zimbabwe and Uganda, the post-crisis governments were a direct continuation of the past, and thus presumably less inclined to undertake drastic reform. The pattern may be different in other countries, like Mozambique, where ‘post-conflict’ came with a new regime.

The important role of development partners and NGOs in post-conflict settings comes as no surprise, but it is interesting to see how their role changes across phases of the post-conflict period, with some roles growing (e.g. funding) while others (e.g. technical support) may tail off as the MoH capacity and confidence grows. The shift from donor dependence is clearly not linear and the timeframes can be longer than suggested by previous literature [42]. The role of the MoH emerges as weaker than expected, even though policy-making processes are generally centralised in all the settings considered. This fits with some earlier studies which suggest that the Ministry of Health is often a low-status ministry in fragile states. It tends to be relatively weak politically, institutionally and financially, with insufficient authority for wider state-building [43]. Moreover, health seems to be more a pre-occupation of the international community than of governments in fragile states. Others highlight unstable “mosaic” policy-making as the prevailing feature, with alliances of actors converging on specific policy issues possessing special appeal at a given point in time, to dissolve quickly as their attention is captured by other concerns [5].

We have examined policies specifically related to HRH issues, which may of course not be typical of all health or other sectoral processes. However, we believe that they are of interest in their own right for a number of reasons. Firstly, HRH is the biggest expenditure item in the health sector and so not just a key input to service delivery but also one of the most important areas for efficiency. Secondly, as HRH offers opportunities for employment and patronage, it is high profile and political. For this reason it is often more closely guarded from external intervention, though as seen in Zimbabwe, donor support as a last resort can be accepted. Third, it is a complex health system pillar because of the important role of human agency, such that HRH policies have to be sophisticated and adaptive. Finally, HRH has implications for other sectors as it influences and is influenced by public sector pay policies.

The features of HRH may explain in part why there seems to be a lot of stickiness in addressing challenges in these settings. Problems are well understood in all four cases but core issues – such as adequate pay, effective distribution and HRH management – are to a greater or lesser degree unresolved. These problems are not confined to post-conflict settings, but underlying challenges to addressing them – including fiscal space, political consensus, willingness to pursue public objectives over private, and personal and institutional capacity to manage technical solutions – are liable to be even more acute in these settings.

## Conclusions

We used mixed research methods to investigate patterns of HRH policy making in four post-conflict and post-crisis settings. HRH was selected as the most expensive, complex and critical health system pillar, and one with more political ramifications. We found that HRH challenges were widely shared across the four cases in the post-conflict period but that the policy trajectories were different – driven by the nature of the conflicts but also the wider context. Windows of opportunity for change and reform can occur but are by no means guaranteed by a crisis – rather they depend on a constellation of leadership, financing, and capacity. Recognition of urgency is certainly a facilitator but not sufficient alone. Post-conflict environments face particularly severe challenges to evidence-based policy making and policy implementation, which also constrain their ability to effectively use the windows which are presented.

## Abbreviations

CBHI: Community-based health insurance
DFID: Department for International Development (UK Aid)
DHO: District Health Officer
FHCI: Free Health Care Initiative (in Sierra Leone)
GDP: Gross domestic product
GMIS: Government midwife incentive scheme (in Cambodia)
HCP: Health Coverage Plan (in Cambodia)
HEF: Health Equity Fund
HRH: Human resources for health
HSSP: Health Sector Strategic Plan
HTF: Health Transition Fund (in Zimbabwe)
KII: Key informant interviews
MoH: Ministry of Health
NGO: Non-governmental organisation
ODs: Operational district (Cambodia)
PBF: Performance-based financing
PNFP: Private not-for-profit
PRDP: Peace Recovery and Development Plan (in Uganda)
RBF: Results-based financing
RDC: Rural District Councils (in Zimbabwe)
ReBUILD: Research for Building Stronger Health Systems Post-Conflict
SOA: Special Operation Agency (in Cambodia)
WHO: World Health Organisation
